# Beyond renal support: biological modulation across pathophysiological domains during continuous renal replacement therapy — a randomized pilot study

**DOI:** 10.3389/fneph.2026.1932419

**Published:** 2026-09-18

**Authors:** Fernando Sánchez-Morán, Salvador Pérez, Juan Sastre-Belloch, María Lidón Mateu-Campos

**Affiliations:** 1 Department of Intensive Care Medicine, General University Hospital of Castellón, Castellón de la Plana, Spain; 2 Department of Medicine, Jaume I University, Castellón de la Plana, Spain; 3 Department of Physiology, Faculty of Pharmacy, University of Valencia, Valencia, Spain

**Keywords:** acute kidney injury, biocompatibility, cell-free DNA, continuous renal replacement therapy, extracorporeal circulation, oxidative stress, regional citrate anticoagulation

## Abstract

**Background:**

Continuous renal replacement therapy (CRRT) is the preferred extracorporeal renal support modality for haemodynamically unstable critically ill patients with acute kidney injury (AKI). Although regional citrate anticoagulation (RCA) is primarily used to maintain circuit patency, its biological effects may extend beyond anticoagulation itself. We hypothesized that different anticoagulation strategies are associated with distinct biological modulation across complementary pathophysiological domains during CRRT.

**Methods:**

We conducted a prospective randomized pilot trial comparing RCA with systemic unfractionated heparin in critically ill adults with AKI requiring CRRT (1:1 allocation). Serial blood samples were obtained before CRRT initiation, at 1 hour and at 24 hours, with paired pre- and post-filter sampling at 1 and 24 hours. Biomarkers represented redox homeostasis (glutathione -GSH, GSSG- and their ratio), neutrophil activation (myeloperoxidase -MPO-), cellular injury (cell-free DNA -cfDNA-) and systemic inflammation (C-reactive protein -CRP-). Healthy reference participants established physiological reference values. Longitudinal analyses used linear mixed-effects models with fixed effects for time, anticoagulation strategy and their interaction. This trial was registered in the European Clinical Trials Database (EudraCT No. 2016-004361-12) and at ClinicalTrials.gov (NCT06646328).

**Results:**

Twenty patients were randomized equally to RCA or systemic heparin; baseline characteristics were comparable between groups. Among six biomarkers spanning four pathophysiological domains, only MPO showed a significant systemic time × anticoagulation interaction (P = .008); although baseline-adjusted sensitivity analyses did not confirm a significant between-group difference. GSH and cfDNA changed significantly over time irrespective of anticoagulation strategy (both P = .016), while the GSSG/GSH ratio approached a treatment-level difference (P = .067). Across the extracorporeal circuit, CRP increased significantly post-filter at both timepoints, MPO increased at 1h, and GSH showed an anticoagulation-dependent pre-to-post change at 1h. No significant differences were observed in secondary clinical outcomes.

**Conclusions:**

These pilot findings suggest that different anticoagulation strategies during CRRT may show domain-specific, rather than uniform, biological modulation, most evident within the neutrophil activation domain, with additional evidence of biomarker-specific kinetics across the extracorporeal circuit. These exploratory results are compatible with the concept that extracorporeal therapies may influence distinct domains differently, a hypothesis warranting confirmation in adequately powered, prospectively designed studies before implications for precision extracorporeal therapy can be drawn.

## 1 Introduction

The evolution of intensive care has been driven not only by technological advances, but also by an increasingly sophisticated understanding of the biological mechanisms underlying critical illness (1–3). As a result, critical illness is increasingly viewed as the consequence of complex and interconnected biological processes driving organ dysfunction rather than as a simple sequence of individual organ failures (4–6). Within this biology-driven framework, oxidative stress has emerged as one of the most extensively investigated biological pathways because of its broad interactions with inflammatory, immune, endothelial and cellular processes involved in critical illness (7).

The unified theory of sepsis-associated acute kidney injury (AKI) (8) represents one of the most compelling examples of this conceptual evolution, proposing that AKI arises from the interaction of inflammatory, microcirculatory, metabolic and cellular pathways rather than from isolated renal hypoperfusion. Within this conceptual framework, severe AKI remains one of the most challenging complications of critical illness, frequently requiring extracorporeal organ support (9). Current international guidelines recommend continuous renal replacement therapy (CRRT) as the preferred renal replacement modality for hemodynamically unstable patients (9, 10). Although conceived as a supportive therapy replacing renal function, CRRT has become an integral component of modern critical care in patients with severe AKI. Its widespread use has also raised new questions beyond its technical performance as a renal support therapy (11).

During CRRT, circulating blood is exposed to artificial surfaces and physicochemical conditions that may influence its biological mileu before returning to the patient. These biological interactions may extend beyond the conventional objectives of renal replacement therapy. Consequently, CRRT may be viewed not solely as a renal support technique, but as a biological intervention whose systemic effects remain incompletely understood (12).

Effective anticoagulation is essential to maintain circuit patency and ensure treatment delivery. Regional citrate and systemic heparin anticoagulation represent the two most widely used anticoagulation strategies during CRRT (13). Within this context, biocompatibility may also be considered in terms of the biological response generated during extracorporeal circulation, rather than solely as the interaction between blood and artificial materials. Consequently, different anticoagulation strategies may contribute differently to this biological response, although the extent to which these differences influence patient biology remains incompletely understood.

Although regional citrate anticoagulation (RCA) was introduced to prevent circuit clotting, it provides a unique opportunity to explore whether its effects extend beyond anticoagulation itself (14). By chelating ionized calcium within the extracorporeal circuit, citrate profoundly modifies the physicochemical environment to which circulating blood is exposed (14, 15). These changes may influence the biological milieu of circulating blood before it returns to the patient.

This perspective provides the rationale for exploring whether different anticoagulation strategies elicit distinct biological responses during CRRT. However, the biological impact of different anticoagulation regimens remains largely unexplored. We therefore hypothesized that RCA and systemic heparin anticoagulation are associated with distinct biological trajectories across complementary pathophysiological domains during CRRT. To explore this hypothesis, we prospectively evaluated representative biomarkers of redox homeostasis, neutrophil activation, systemic inflammation and cellular injury in a randomized pilot study of critically ill patients with AKI requiring CRRT (**Figure 1**).

**Figure 1:**
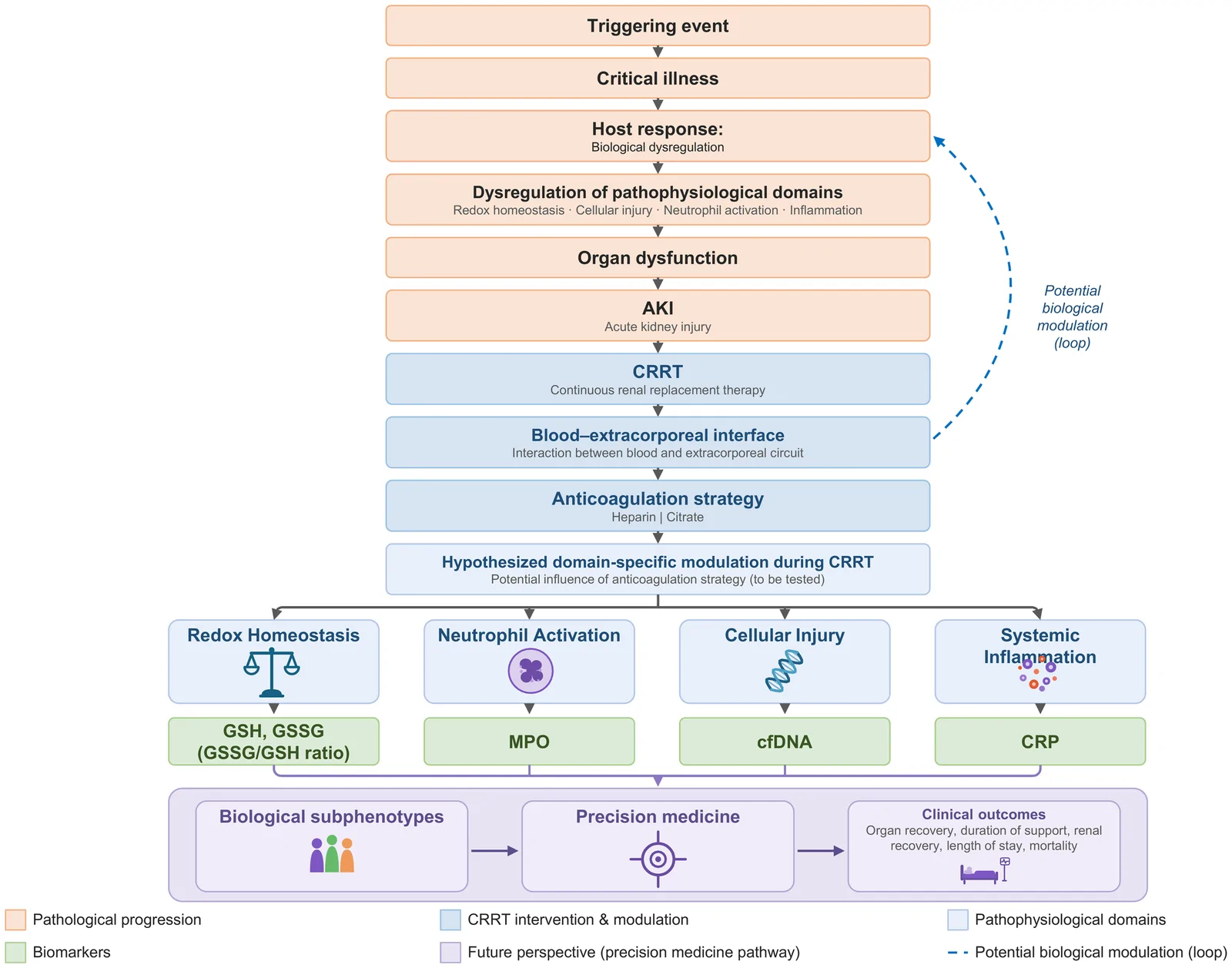
Conceptual framework. Conceptual framework linking critical illness, host biological dysregulation, acute kidney injury and CRRT. The hypothesized influence of anticoagulation strategy on domain-specific biological modulation, and the potential feedback loop toward host response, are shown as conceptual/hypothesized elements to be tested by the present study, not as established findings. AKI: acute kidney injury; CRRT: continuous renal replacement therapy.

## 2 Methods

### 2.1 Study design

This investigator-initiated, prospective, randomized, parallel-group pilot trial was conducted between June 2018 and May 2022 in the intensive care units of the General University Hospital of Castellón and the University Hospital of La Plana, Spain. Adult critically ill patients with AKI requiring CRRT were randomly assigned in a 1:1 ratio to receive either regional citrate anticoagulation (RCA) or systemic unfractionated heparin anticoagulation.

The random allocation sequence was generated using a computer-based randomization program, using permuted blocks of four patients (two allocated to each treatment group per block) and without stratification by study centre, before study initiation. Allocation concealment was ensured using sequentially numbered, opaque, sealed envelopes prepared by an investigator not involved in patient recruitment or treatment allocation. Randomization was performed immediately before CRRT initiation after confirmation that all eligibility criteria had been fulfilled and written informed consent had been obtained.

### 2.2 Ethics statement

The study was authorised by the Spanish Agency of Medicines and Medical Devices (AEMPS) as a clinical trial and conducted in accordance with the Declaration of Helsinki (16) and Good Clinical Practice guidelines. It was registered in the European Clinical Trials Database (EudraCT No. 2016-004361-12) and at ClinicalTrials.gov (NCT06646328). The original protocol was approved by the Research Ethics Committee (CEI) of University Hospital of La Plana on 2 March 2017 (Act 2-2017). A substantial protocol amendment, approved in 2019, incorporated the General University Hospital of Castellón as a second participating centre. The study was subsequently approved by the Clinical Research Ethics Committee of the General University Hospital of Castellón (Approval Act 7/2019) and conducted in accordance with the original protocol and all approved amendments. Written informed consent was obtained from all participants or, when patients lacked decision-making capacity, from their legally authorised representatives before enrolment.

### 2.3 Study population

Adult critically ill patients admitted to the participating intensive care units who developed AKI requiring CRRT, as determined by the treating intensivist according to clinical judgment, were screened for eligibility. AKI was defined according to Kidney Disease: Improving Global Outcomes (KDIGO) criteria (10). Patients meeting all inclusion criteria and none of the predefined exclusion criteria were consecutively enrolled after written informed consent had been obtained from the patient or, when the patient lacked decision-making capacity, from a legally authorised representative.

#### 2.3.1 Inclusion criteria

- Age 18–80 years.
- Admission to the intensive care unit.
- AKI requiring CRRT.
- Written informed consent obtained from the patient or, when applicable, from a legally authorized representative.

#### 2.3.2 Exclusion criteria

Patients meeting any of the following criteria were excluded:

- Pregnancy or breastfeeding.
- Terminal illness or expected survival <48 hours.
- Increased bleeding risk, defined as a platelet count <40 × 10^9^/L, activated partial thromboplastin time (aPTT) >60 s, international normalized ratio (INR) >2.0, or recent major bleeding.
- Requirement for therapeutic systemic anticoagulation.
- Contraindication to unfractionated heparin.
- History of heparin-induced thrombocytopenia.
- Renal replacement therapy within the previous 24 hours.
- Hypercalcaemia (serum calcium >3 mmol/L).
- Severe acute hepatitis (AST or ALT >1000 IU/L).
- Liver cirrhosis.
- Participation in another interventional clinical trial.

### 2.4 CRRT and anticoagulation protocol

CRRT initiation criteria included oliguria or anuria associated with diuretic-refractory fluid overload, or AKI associated with metabolic acidosis (pH <7.25), hyperkalaemia (>6 mmol/L), or serum urea >120 mg/dL. Renal support was discontinued after recovery of spontaneous urine output (>700 mL/day without diuretics), absence of fluid overload, resolution of metabolic disturbances, and creatinine clearance >20 mL/min. All decisions regarding CRRT initiation and discontinuation were made by the treating intensivist without investigator involvement.

All patients received CRRT in continuous veno-venous haemodialysis (CVVHD) mode. Nineteen patients were treated using the multiFiltrate^®^ platform with an Ultraflux^®^ EMiC^®^2 filter (Fresenius Medical Care, Bad Homburg, Germany), while one patient received treatment using the Prismaflex^®^ platform with an AN69ST (ST150) filter (Baxter Healthcare, Deerfield, IL, USA). Vascular access was obtained through a double-lumen catheter inserted into the femoral or internal jugular vein. Patients allocated to the RCA group received a 4% trisodium citrate solution (136 mmol/L citrate; Fresenius Medical Care) infused into the arterial limb of the extracorporeal circuit before the filter. A calcium-free citrate-adapted dialysate (Ci-Ca^®^ Dialysate K4; sodium 133 mmol/L, potassium 4 mmol/L, bicarbonate 20 mmol/L, calcium 0 mmol/L) was used throughout treatment. Systemic calcium replacement was achieved by continuous post-filter infusion of calcium chloride (97 mmol/L). Systemic ionized calcium concentrations were maintained between 1.12 and 1.20 mmol/L, while post-filter ionized calcium was targeted between 0.25 and 0.35 mmol/L, according to the institutional protocol. Ionized calcium measurements were routinely performed every six hours, and treatment adjustments were made by the attending clinical team.

Patients randomized to systemic anticoagulation received unfractionated heparin administered through the arterial limb of the extracorporeal circuit before the blood pump. Standard calcium-containing dialysate (MultiBic^®^ 4 mmol/L potassium or Prismasol^®^ 4, according to the CRRT platform employed) was used. Heparin dosing and monitoring followed the routine institutional anticoagulation protocol.

Except for the anticoagulation strategy and the corresponding dialysis solutions required for RCA, all CRRT prescriptions followed routine institutional practice and were independent of study participation. Additional technical details regarding the CRRT and anticoagulation protocols are provided in **Supplementary Methods**.

### 2.5 Sample collection

Blood samples were obtained at three predefined time points: immediately before CRRT initiation (T0), one hour after treatment initiation (T1), and twenty-four hours after treatment initiation (T2). At T1 and T2, paired blood samples were simultaneously collected from the pre-filter and post-filter ports of the extracorporeal circuit. Effluent samples were also collected to explore the potential extracorporeal transfer of the investigated biomarkers. The original protocol specified the intermediate sampling time point (T1) at 6 hours after CRRT initiation. In practice, T1 sampling was performed at 60 minutes (1 hour) after initiation in all 20 participants, a deviation reflecting a pragmatic operational constraint (as only a single investigator was trained in sample processing at the start of recruitment), and it was applied consistently throughout the study, ensuring standardized sample collection and processing.

Immediately after collection, blood samples were placed on ice and transported to the laboratory. Sample processing began within approximately 10 minutes of collection. Plasma was separated by centrifugation according to standardized laboratory procedures, aliquoted, and stored at −80 °C within approximately 40 minutes of blood collection.

Because recruitment extended over several years, GSH and GSSG were analysed in two analytical batches corresponding to the first and second halves of the recruited cohort. MPO and cfDNA were each analysed in a single analytical batch after completion of recruitment. All biomarker analyses were performed by an external university laboratory according to standardized operating procedures. For GSH, GSSG and cfDNA, the laboratory reported the final concentration values. For MPO, raw analytical output was additionally archived as part of the study documentation.

### 2.6 Biomarker analysis

Biomarkers were selected *a priori* to represent complementary pathophysiological domains potentially involved in the biological response of critical illness and extracorporeal circulation. The redox homeostasis domain was evaluated through circulating concentrations of reduced glutathione (GSH), oxidized glutathione (GSSG) and the calculated GSSG/GSH ratio. The neutrophil activation domain was represented by plasma myeloperoxidase (MPO) activity. The cellular injury domain was evaluated through circulating cell-free DNA (cfDNA) concentrations. The systemic inflammation domain was assessed by serum C-reactive protein (CRP), measured by the hospital’s routine clinical chemistry laboratory using a standard immunoturbidimetric assay.

Given the nature of the interventions, clinicians and patients could not be blinded to anticoagulation strategy; however, laboratory personnel performing biomarker analyses were blinded to treatment allocation throughout sample measurement.

#### 2.6.1 Reduced and oxidized glutathione

Whole blood samples for GSH and GSSG were collected in ethylenediaminetetraacetic acid (EDTA) tubes and processed immediately. To 250 μL of blood, 150 μL of phosphate-buffered saline and 100 μL of 30% perchloric acid were added; samples were centrifuged (15,000 × g, 15 min) and the supernatant stored at −80 °C until analysis. GSH and GSSG were quantified by liquid chromatography–tandem mass spectrometry, using a Micromass Quattro™ triple-quadrupole mass spectrometer with a Z-spray ionization source operated in positive-ion mode, coupled to a Shimadzu LC-10A HPLC system and MassLynx 4.1 software. Reverse-phase separation used a Mediterranean Sea C18 column (5.0 × 0.21 cm, 3 μm particle size; Teknokroma, Barcelona, Spain), with a 20 μL injection volume, a gradient mobile phase (A: 0.5% formic acid; B: isopropanol/acetonitrile 50/50 with 0.5% formic acid) at 0.2 mL/min, nitrogen as drying/nebulizing gas, and argon as collision gas. Six-point calibration curves (0.01–100 μmol/L; Sigma-Aldrich, Missouri, USA) were used for quantification. Raw assay output is expressed in μmol/mL for GSH and nmol/mL for GSSG; for consistency with conventional clinical reporting, GSH concentrations are presented as μmol/L (raw μmol/mL values multiplied by 1000) throughout the Results, Tables and Figures, while GSSG concentrations in nmol/mL are numerically equivalent to μmol/L and are presented as such without further transformation. The GSSG/GSH ratio is reported ×1000 for readability, given that GSSG concentrations are physiologically much lower than GSH.

#### 2.6.2 Myeloperoxidase

MPO activity was determined by spectrophotometry in plasma from EDTA whole blood stored at −80 °C. The assay is based on peroxidase-catalysed oxidation of tetramethylbenzidine (TMB) in the presence of hydrogen peroxide, with the reaction monitored spectrophotometrically. Enzyme extraction used monopotassium phosphate buffer (pH 6) with plasma and buffer mixed at a 1:1 (v/v) ratio, followed by centrifugation (20,000 × g, 20 min, 4 °C) to separate MPO from other peroxidases. The resulting sedimented fraction (pellet) was resuspended in monopotassium phosphate buffer (pH 6) with hexadecyltrimethylammonium bromide (a quaternary ammonium detergent) to disrupt granules. According to the laboratory protocol, HTAB treatment, combined with three cycles of sonication and freeze-thawing, was used to solubilize the particulate/granular fraction and disrupt granules before the final centrifugation (15,000 × g, 15 min, room temperature). The peroxidase reaction (TMB followed by hydrogen peroxide) was performed in 96-well plates and monitored kinetically at 620 nm using ten sequential readings at 10-second intervals. Activity is expressed as mU/mL (one unit = amount of TMB oxidized per minute), referenced against a standard peroxidase curve (Sigma-Aldrich, Missouri, USA; assay range 1.5–100 mU/mL).

Quality control was assessed from the archived raw analytical output. The assay included ten sequential kinetic readings per well at 10-second intervals. All seven calibration standards showed highly linear kinetic responses (R² range, 0.9974–0.9994), and the cubic calibration curve generated from the calculated kinetic rates showed an R² of 0.99989. MPO was analyzed in a single analytical session; therefore, inter-assay or inter-batch precision could not be estimated, and replicate-based intra-assay coefficients of variation were not available.

The peroxidase assay was performed and read by a laboratory technician blinded to treatment allocation. A study investigator was present solely to record the correspondence between microplate well position and coded sample identifier, for subsequent linkage to the study database; this investigator did not perform or interpret the assay and was not aware of treatment allocation at the time of sample loading, so that blinding of the analytical result was maintained.

#### 2.6.3 Cell-free DNA

cfDNA was quantified by fluorometry. Whole blood was collected in EDTA tubes and processed immediately by centrifugation (200 × g, 15 min); plasma was stored at −80 °C until analysis. Fifty μL of plasma were added to 96-well plates with 50 μL of SYTOX Green (Molecular Probes; 1 μM in PBS), a fluorescent nucleic-acid-binding dye, and plates were read immediately on a fluorescence plate reader at 485/528 nm excitation/emission. Results are expressed in ng/mL.

### 2.7 Outcomes

The registered clinical primary outcome was a composite of days on renal replacement therapy, renal function at ICU and hospital discharge, and status at 30 days after ICU discharge (renal function, dialysis dependence, and survival). Among the study’s biological objectives, the original protocol designated oxidative stress (GSH, GSSG) and circulating cell-free DNA as primary academic objectives, with neutrophil activation (MPO) and systemic inflammation (CRP) as secondary biological objectives. The protocol’s formal evaluation criteria subsequently listed GSH, GSSG, the GSSG/GSH ratio and MPO together, without further hierarchy, as secondary evaluation criteria. This manuscript reports and interprets the biological domains as its principal focus, given that the achieved sample size was recognised, ahead of analysis, to be underpowered for the registered clinical primary outcome; all six biomarker comparisons are treated as exploratory and hypothesis-generating on an equal footing, and none is presented as a confirmatory primary outcome.

Secondary clinical outcomes included renal recovery, duration of CRRT, Intensive Care Unit (ICU) and hospital length of stay, and ICU and hospital mortality. As an additional, exploratory outcome, we assessed biomarker removal/generation across the extracorporeal circuit, by comparing paired pre- and post-filter concentrations at 1h and 24h.

### 2.8 Healthy reference participants

Healthy reference participants were prospectively recruited to establish physiological reference values for the evaluated biomarkers. Recruitment was performed after completion of patient enrolment, between 6 and 9 June 2022 (n = 8), among ambulatory adults undergoing elective digestive endoscopy under procedural sedation. Participants were selected to achieve an age and sex distribution broadly comparable to that of the study cohort, without individual matching. Blood samples were obtained through the peripheral venous catheter routinely placed for the procedure, without additional intervention. This cohort was not intended as a clinical control group but solely to provide physiological reference values for interpretation of biomarker measurements obtained during CRRT.

### 2.9 Statistical analysis

#### 2.9.1 Sample size

The original trial protocol (version 2.0, 13 January 2017) specified a pragmatic sample size target of 44 randomized patients (20 per arm, inflated to 22 per arm assuming 10% attrition), based on the sample size of comparable pilot studies rather than a formal power calculation, alongside a parallel cohort of 22 healthy reference participants. In October 2019, with recruitment slower than anticipated, the study team incorporated a literature-based power calculation for MPO, the only biomarker in the panel for which comparable published data allowing such a calculation were available, using the study by Bos et al. (17): assuming a difference in MPO concentrations of 220 mU/mL between baseline and 180 minutes, with a standard deviation of 126 mU/mL, an α error of 0.05 and a statistical power of 90%, the estimated core sample size was 18 patients, inflated by 20% (to 22 patients) to accommodate anticipated losses to follow-up. This calculation, based on a 180-minute comparison, was applied as the best available proxy for the study’s own sampling schedule (60 minutes/1h and 24h); at the time, this was the only published reference the investigators had identified reporting MPO changes under heparin versus citrate anticoagulation. The final analyzed sample of 20 patients exceeds the calculated minimum sample size (18 patients), though it falls short of the loss-inflated target (22 patients), consistent with attrition being more limited than originally anticipated. Recruitment was subsequently fragmented, rather than halted at a single decision point, by successive COVID-19 pandemic waves between 2020 and 2022, with intermittent pauses during peak periods and partial resumption during lower-intensity phases. This substantially extended the overall recruitment timeline. Recruitment concluded in 2022 with a final analyzed sample of 20 patients, close to, though not the result of a deliberate decision to stop upon reaching, the October 2019 literature-based target. Neither this revised sample-size justification nor the biomarker on which it was based was specified as the study’s prospective primary outcome (see Section 2.7); biomarker analyses were therefore considered exploratory and hypothesis-generating throughout, and MPO is best regarded as the only biomarker for which a subsequent, literature-based power justification was incorporated once the study was already underway, rather than as a prospectively powered primary biological outcome.

Continuous variables are presented as mean ± standard deviation or median (interquartile range), according to data distribution. Categorical variables are expressed as frequencies and percentages.

Normality was assessed using the Shapiro–Wilk test. Between-group comparisons were performed using Student’s t-test or the Mann–Whitney U test, as appropriate. Categorical variables were compared using the χ² test or Fisher’s exact test.

Analytical outliers, identified by standardized z-scores (|z|>2 SD from the study mean), were excluded from longitudinal analyses according to a criterion defined by the study team prior to data analysis; this criterion was not documented in a separate, formal statistical analysis plan, consistent with the original protocol’s specification of intended methods in general terms rather than a detailed prespecified analysis plan (**Supplementary Table 1**). Results without this exclusion are reported alongside the analysis with outlier exclusion as a sensitivity check (Section 3.2; **Supplementary Table 3**).

The longitudinal analyses were performed using linear mixed-effects models including fixed effects for time, anticoagulation strategy and the time × anticoagulation interaction, with patient identity incorporated as a random effect to account for repeated measurements. Statistical inference for fixed effects was based on Satterthwaite-approximated degrees of freedom. Likelihood-ratio χ^2^ tests for the same fixed effects, used in sensitivity analyses (**Supplementary Table 3**) and in response to reviewer comments, are reported alongside the omnibus F-tests where relevant. Extracorporeal biomarker kinetics were assessed by comparing paired pre- and post-filter concentrations on each occasion using linear mixed-effects models with fixed effects for filter position, anticoagulation strategy and their interaction, and patient identity as a random intercept. Linear mixed-effects models were selected because they appropriately account for the correlation between repeated measurements within individuals while allowing inclusion of all available longitudinal observations.

Baseline-adjusted sensitivity analyses were also performed using analysis of covariance (ANCOVA), with baseline biomarker values included as a covariates. ANCOVA was performed using the analytical dataset after application of the predefined outlier criterion; corresponding adjusted coefficients and 95% confidence intervals are provided in **Supplementary Table 6**.

Flow-related influences on the paired pre- and post-filter concentrations were quantified from the prescribed circuit settings. For citrate-treated patients, the expected haemodilution at the pre-filter sampling port was estimated from the prescribed blood flow and citrate infusion rate. The filtration fraction was estimated for each patient as the ultrafiltration rate divided by the incoming plasma flow (blood flow × [1 − haematocrit]), and the corresponding post-filter haemoconcentration factor as 1/(1 − filtration fraction). Because these estimates derive from prescribed rather than directly measured flows, and because haematocrit was an estimated value in 12 of the 20 patients, they are reported as order-of-magnitude benchmarks and were not used to adjust the reported concentrations, with one exception: in a sensitivity analysis, citrate-group MPO concentrations at 1 h and 24 h were corrected for the estimated dilution and the time × treatment interaction was re-estimated.

To evaluate and operationalize the composite clinical outcome conceptually outlined in the original study protocol (incorporating survival, renal recovery, and CRRT duration), we constructed a hierarchical ordinal clinical score ranging from 0 (worst) to 4 (best) following contemporary critical care research methodology. Each patient’s clinical trajectory was ranked by hierarchically integrating: (1) hospital survival, (2) non-dialysis dependency and renal function at hospital discharge, and (3) cumulative CRRT duration (using total days of therapy). Scores were assigned as follows: Score 0, in-hospital mortality with CRRT duration at or below the overall study mean (3.0 days); Score 1, in-hospital mortality with CRRT duration above the study mean; Score 2, survival to hospital discharge with negative renal recovery (persistent dialysis dependency or creatinine increase >50% from baseline); Score 3, survival with positive renal recovery (non-dialysis dependent, creatinine rise <50% from baseline) but CRRT duration above the study median (2.5 days); Score 4, survival with positive renal recovery and CRRT duration at or below the study median. Distribution of this composite score between anticoagulation strategies was compared using the Mann–Whitney U test. Although the composite outcome was conceptually defined in the original protocol, the ordinal scoring structure applied here was constructed for the present analysis and was not prespecified in the 2017 protocol; it should therefore be regarded as a *post hoc* operationalization of that outcome.

Statistical significance was established at a two-sided P value <0.05 for all tests. Statistical analyses were performed using IBM SPSS Statistics version 29.0.2.0 (IBM Corp., Armonk, NY, USA) and jamovi version 2.7.

Pairwise comparisons between individual time points, by anticoagulation group, are presented in **Supplementary Table 4**.

## 3 Results

### 3.1 Patient enrolment and baseline characteristics

Between June 2018 and May 2022, 227 critically ill patients with AKI receiving CRRT were screened, of whom 20 were ultimately randomized to receive RCA (n=10) or systemic unfractionated heparin (n=10); reasons for exclusion and non-enrolment are detailed in the patient flow diagram (**Figure 2**).

**Figure 2:**
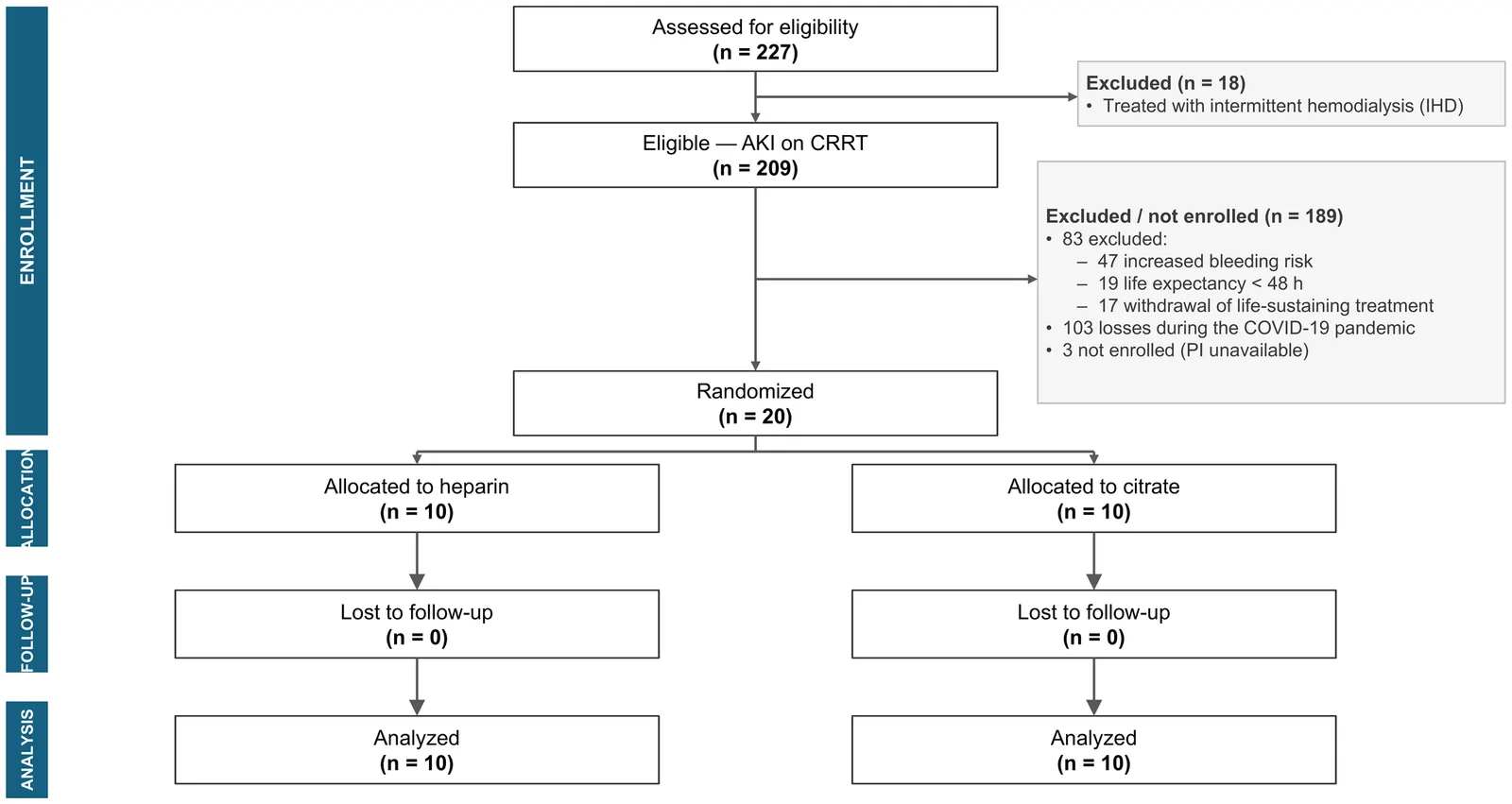
Patient flow diagram. Flow diagram of patient screening, exclusion, randomization and analysis. AKI, acute kidney injury; CRRT, continuous renal replacement therapy; IHD, intermittent haemodialysis; PI, principal investigator.

Baseline demographic characteristics, illness severity, indications for CRRT, clinical status at CRRT initiation and prescribed extracorporeal treatment were comparable between treatment groups (**Table 1**). No clinically relevant differences were observed in age, sex, illness severity scores, renal dysfunction at laboratory variables or CRRT prescription. By design, because citrate solution was administered as pre-filter replacement fluid, total convective dose was substantially higher in the citrate group (3.02 [IQR 1.98–4.15] mL/kg/h) than in the heparin group (0.54 [0–0.94] mL/kg/h; P<.001, Cohen’s d=2.78), reflecting the anticoagulation method itself rather than a difference in prescribed renal support intensity.

**Table 1:** Baseline characteristics at ICU admission and at initiation of CRRT by anticoagulation strategy, and treatment parameters at initiation of CRRT.

Characteristic	Global (n=20)	Heparin (n=10)	Citrate (n=10)	P
Demographics				
Age, years	64.20 (± 9.92)	60.87 (± 11.11)	68.55 (± 7.32)	.085
Male sex, n (%)	17 (85)	7 (70)	10 (100)	.211
Comorbidities				
Ischaemic heart disease, n (%)	3 (15)	1 (10)	2 (20)	1.000
Dyslipidaemia, n (%)	10 (50)	5 (50)	5 (50)	1.000
Diabetes mellitus, n (%)	5 (25)	3 (30)	2 (20)	1.000
Hypertension, n (%)	13 (65)	6 (60)	7 (70)	1.000
Obesity, n (%)	6 (30)	4 (40)	2 (20)	.628
COPD, n (%)	3 (15)	1 (10)	2 (20)	1.000
Chronic kidney disease, n (%)	3 (15)	2 (20)	1 (10)	1.000
At ICU admission				
APACHE II	18.90 (± 5.87)	18.40 (± 7.63)	19.40 (± 3.72)	.714
SAPS 3	64.15 (± 14.55)	62.30 (± 16.25)	66.00 (± 13.24)	.584
SOFA	8.75 (± 2.40)	8.00 (6.75–12.25)	9.00 (7.00–9.00)	.912
Mechanical ventilation, n (%)	10 (50)	5 (50)	5 (50)	1.000
Invasive/non-invasive, n (%)	9/1 (45/5)	4/1 (40/10)	5/0 (50/0)	1.000
FiO_2_, %	50 (40–53)	50 (48–75)	40 (38–55)	.195
Vasopressors, n (%)	17 (85)	8 (80)	9 (90)	1.000
Noradrenaline dose, µg/kg/min	0.37 (± 0.29)	0.30 (± 0.32)	0.44 (± 0.25)	.274
Lactate, mmol/L	2.20 (1.35–4.43)	2.30 (1.35–4.10)	1.90 (1.20–6.90)	.930
Urea, mg/dL	77 (56–193)	122.33 (± 91.92)	127.90 (± 98.41)	.900
Creatinine, mg/dL	3.59 (± 2.20)	3.70 (± 2.14)	3.48 (± 2.38)	.836
Bilirubin, mg/dL	0.86 (0.49–2.16)	1.33 (0.92–2.48)	0.54 (0.34–2.54)	.102
Leukocytes, ×10³/µL	12.24 (8.31–16.83)	12.80 (8.25–14.78)	11.69 (7.88–28.18)	.796
Platelets, ×10³/mm³	180.00 (142.50–312.00)	179.00 (134.50–221.25)	228.50 (160.50–332.50)	.290
At initiation of CRRT				
Mechanical ventilation, n (%)	10 (50)	6 (60)	4 (40)	1.000
Invasive/non-invasive, n (%)	7/3 (35/15)	4/2 (40/20)	3/1 (30/10)	1.000
FiO_2_, %	50 (35–60)	53 (50–63)	35 (28–70)	.195
Vasopressors, n (%)	19 (95)	9 (90)	10 (100)	1.000
Noradrenaline dose, µg/kg/min	0.45 (0.29-0.70)	0.40 (0.09-0.95)	0.45 (0.20-0.70)	.677
Lactate, mmol/L	1.80 (1.00–3.20)	1.80 (1.08–3.15)	1.75 (0.78–4.28)	.910
Urea, mg/dL	132 (69.50–194.50)	127.00 (67.75-174.25)	154.00 (76.50-200.50)	.900
Creatinine, mg/dL	4.11 (2.69-5.80)	4.92 (2.30-6.02)	3.78 (2.77-4.60)	.545
Bilirubin, mg/dL	0.58 (0.34–1.49)	1.11 (0.46–1.73)	0.47 (0.33–1.57)	.102
Leukocytes, ×10³/µL	14.16 (7.88–24.07)	14.16 (10.03–18.17)	15.19 (6.66–31.35)	.796
Platelets, ×10³/mm³	182.00 (149.75–303.00)	169.75 (132.00–254.25)	241.00 (161.75–314.00)	.290
CRRT initiation parameters				
Blood flow, ml/min	100 (100–127.50)	100 (100-127.50)	100 (100-132.50)	.723
Dialysate flow, ml/h	2000 (2000-2575)	2000 (1937-2625)	2000 (2000-2650)	.902
Dialysis dose, ml/kg/h	25.00 (19.71-30.77)	25.00 (18.35-31.66)	25.04 (19.36-31.70)	.969
Ultrafiltration rate, ml/h	50 (0-100)	50 (0-100)	25 (0-100)	.630
Ultrafiltration dose, ml/kg/h	0.48 (0-0.92)	0.54 (0-0.94)	0.23 (0-1.00)	.751
Citrate convection dose, ml/kg/h	0 (0-2.54)	0	2.54 (1.82-4.15)	<.001
Total convection dose, ml/kg/h	1.43 (0.52-3.08)	0.54 (0-0.94)	3.02 (1.98-4.15)	<.001
Initial total dose, ml/kg/h	27.58 (19.83-34.72)	25.63 (18.56-31.83)	29.86 (21.33-39.25)	.481

Qualitative variables shown as n (%); quantitative variables as mean (± SD) or median (IQR). SD, standard deviation; IQR, interquartile range; COPD, chronic obstructive pulmonary disease; FiO_2_, fraction of inspired oxygen. Values correspond to ICU admission. APACHE II, Acute Physiology and Chronic Health Evaluation II; SAPS 3, Simplified Acute Physiology Score 3; SOFA, Sequential Organ Failure Assessment; ICU, intensive care unit.

All randomized patients contributed biological samples and were included in the longitudinal analyses. Healthy reference cohort comprised 8 participants.

### 3.2 Systemic biological trajectories by pathophysiological domain

Longitudinal analyses were performed using mixed-effects model including fixed effects for time, anticoagulation strategy and their interaction, with patient identity as a random intercept, on pre-filter (systemic) biomarker concentrations. Analytical outliers (|z| > 2 SD) were excluded per the criterion described in Section 2.9. Across the six biomarkers spanning four pathophysiological domains, the time × anticoagulation interaction reached statistical significance only for MPO (neutrophil activation domain; P = .008) (**Table 2**). Because baseline (T0) MPO concentrations differed descriptively between groups, baseline-adjusted ANCOVA models were additionally fitted at each follow-up timepoint (**Supplementary Table 6**). After adjustment for baseline values, between-group differences were not statistically significant at T1 (β=14.4 mU/mL [95% CI −13.7, 42.6], P = .29, n=19, identical with and without exclusion, as no T0 or T1 outliers were identified for MPO) or T2 (β=8.4 mU/mL [95% CI −19.5, 36.2], P = .53, n=15, with outlier exclusion; β=22.3 [−12.6, 57.0], P = .19, n=17, without exclusion). GSH and cfDNA concentrations changed significantly over time irrespective of anticoagulation strategy, while the GSSG/GSH ratio showed a treatment-level difference approaching, but not reaching, conventional significance. Longitudinal trajectories for all six biomarkers are shown in **Figure 3**.

**Table 2:** Linear mixed-effects analyses of pre-filter biomarker trajectories, by pathophysiological domain.

Domain	Biomarker	n (obs/pts)	Time effect, F, P	Anticoagulation effect, F, P	Time × Anticoagulation, F, P	Δ(T1−T0)×Tx β [95% CI]	Δ(T2−T0)×Tx β [95% CI]	ICC
Redox homeostasis								
	GSH	56/20	F(2,33.1)=4.72, P = .016	F(1,18.2)=1.97, P = .178	F(2,33.1)=0.34, P = .712	-7.87 [-44.06, 28.30]	8.14 [-30.86, 47.10]	41.3%
	GSSG	54/19	F(2,29.9)=0.51, P = .608	F(1,14.9)=0.32, P = .578	F(2,29.9)=0.93, P = .407	-0.34 [-0.86, 0.17]	-0.23 [-0.77, 0.31]	25.6%
	GSSG/GSH ratio	51/19	F(2,45.0)=0.55, P = .583	F(1,45.0)=3.53, P = .067	F(2,45.0)=0.65, P = .525	-1.50 [-4.39, 1.40]	-1.41 [-4.48, 1.67]	0.0%
Neutrophil activation								
	**MPO**	**53/19**	**F(2,31.1)=1.33, P = .279**	**F(1,17.5)=2.24, P = .152**	**F(2,31.1)=5.68, P = .008**	**33.14 [4.75, 61.54]**	**49.46 [18.59, 80.32]**	**54.0%**
Cellular injury								
	cfDNA	53/20	F(2,29.7)=4.79, P = .016	F(1,17.5)=1.60, P = .222	F(2,29.7)=0.37, P = .696	-114.50 [-561.00, 332.00]	94.00 [-404.00, 592.00]	67.2%
Systemic inflammation								
	CRP	53/20	F(2,29.2)=1.27, P = .295	F(1,17.8)=3.78, P = .068	F(2,29.2)=0.23, P = .797	25.61 [-51.40, 102.60]	9.19 [-76.90, 95.30]	86.0%

Model: Biomarker ~ Time + Anticoagulation strategy + Time × Anticoagulation strategy, patient identity as random intercept (|z|>2 SD excluded per an analytic criterion defined by the study team prior to data analysis, not documented in a separate formal statistical analysis plan). Time and Anticoagulation main-effect P-values shown here derive from omnibus F-tests and Satterthwaite-approximated degrees of freedom; corresponding likelihood-ratio χ^2^ tests are reported in **Supplementary Table 3** and differ numerically from those shown here, including for the Time x Anticoagulation interaction in most biomarkers. For MPO the Time x Anticoagulation interaction P-value is numerically identical between both approaches (P = .008). Omnibus F-tests and Satterthwaite degrees of freedom computed in jamovi (GAMLj module, lme4 backend). Δ(T1−T0)×Tx and Δ(T2−T0)×Tx are the anticoagulation-coded (reference T0) interaction coefficients, the between-group difference in the change from baseline to 1h and from baseline to 24h, respectively (Citrate minus Heparin), with 95% confidence intervals. The MPO row denotes the only significant time × anticoagulation interaction (P<0.05). obs, observations; pts, patients; CI, confidence interval; Tx, anticoagulation strategy (treatment); ICC, intraclass correlation coefficient. GSH, reduced glutathione; GSSG, oxidized glutathione; MPO, myeloperoxidase; cfDNA, cell-free DNA; CRP, C-reactive protein. Bold indicates P < .05.

**Figure 3:**
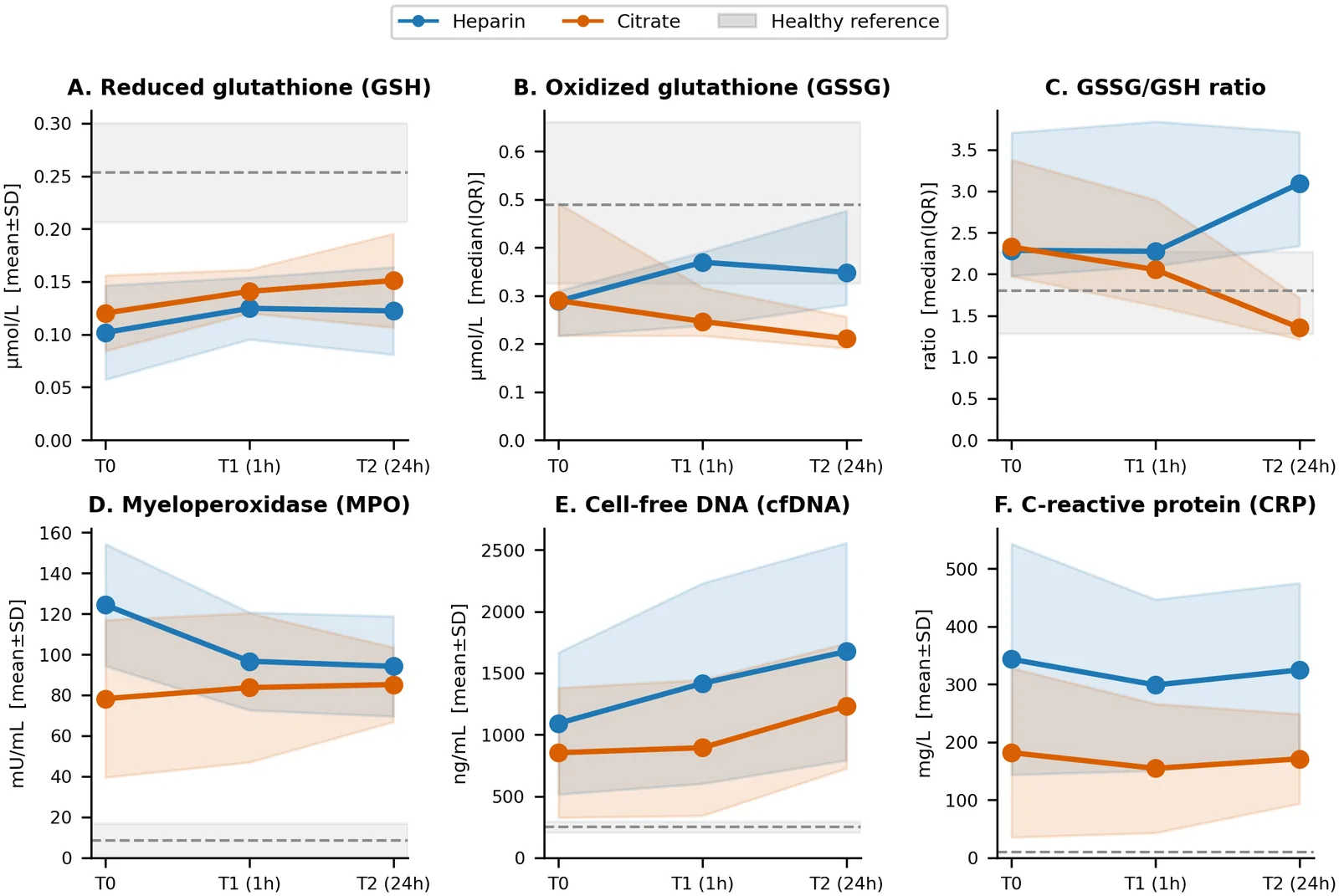
Longitudinal biomarker trajectories by anticoagulation strategy. Longitudinal trajectories of pre-filter **(A)** reduced glutathione, **(B)** oxidized glutathione, **(C)** GSSG/GSH ratio, **(D)** myeloperoxidase, **(E)** cell-free DNA and **(F)** C-reactive protein, by anticoagulation strategy. The x-axis represents the three sampling time points: T0, T1 (1 hour after T0) and T2 (24 hours after T0). Lines/shading represent mean ± SD or median(IQR) per group (selected per-domain according to Shapiro-Wilk normality); dotted lines mark the upper/lower bounds of each band. Bands and axes are truncated at zero to reflect the biological non-negativity of the measured quantities. Grey dashed line/band: healthy reference cohort (baseline only); for CRP, the standard clinical upper limit of normal (10 mg/L) is shown instead. Outliers (|z| > 2 SD) were excluded, consistent with the criteria applied in **Table 2**.

### 3.3 Secondary outcome: clinical outcomes

No statistically significant differences were observed between the RCA and systemic heparin groups in duration of CRRT (median 2.00 [IQR 1.00–3.50] vs 3.50 [2.00–8.00] days; P = .114), ICU length of stay (4.50 [1.75–7.75] vs 8.50 [3.75–12.25] days; P = .165), hospital length of stay (19.50 [12.75–30.75] vs 18.50 [12.00–48.75] days; P = .853), or renal recovery at ICU discharge (5/7 [71.4%] vs 2/6 [33.3%] with a positive renal outcome; P = .286, Fisher’s exact test) or at hospital discharge (6/6 [100%] vs 5/6 [83.3%]; P = 1.000) (**Table 3**). ICU mortality was 40% (4/10) in the heparin group and 30% (3/10) in the citrate group (Fisher’s exact P = 1.000); hospital mortality was 40% (4/10) in both groups (P = 1.000). In an exploratory hierarchical composite analysis integrating hospital survival, renal function at discharge, and cumulative CRRT days conceptually defined in the study protocol, into a single global clinical score (0–4 scale), no significant difference was observed between treatment arms (U = 47.0, p=.846; per-patient score derivation in **Supplementary Table 7**). Given the exploratory nature of these analyses, the small sample size and the fact that the study was not powered for clinical endpoints, these comparisons should be interpreted as descriptive and hypothesis-generating rather than confirmatory.

**Table 3:** Secondary clinical outcomes by anticoagulation strategy.

Outcome	Heparin (n=10)	Citrate (n=10)	P/note
Duration of CRRT, days, median (IQR)	3.50 (2.00–8.00)	2.00 (1.00–3.50)	P=.114
ICU length of stay, days, median (IQR)	8.50 (3.75–12.25)	4.50 (1.75–7.75)	P=.165
Hospital length of stay, days, median (IQR)	18.50 (12.00–48.75)	19.50 (12.75–30.75)	P=.853
Renal recovery at ICU discharge, n (%)^1^	2/6 (33.3)	5/7 (71.4)	P=.286
Renal recovery at hospital discharge, n (%)^1^	5/6 (83.3)	6/6 (100.0)	P=1.000
ICU mortality, n (%)	4 (40.0)	3 (30.0)	P=1.000
Hospital mortality, n (%)	4 (40.0)	4 (40.0)	P=1.000
Hierarchical composite clinical score (0–4), median (IQR)	2.50 (1.00–3.00)	3.00 (0.25-4.00)	U=47.0, P = .846

^1^ Renal recovery = positive outcome (preserved/recovered renal function, defined as creatinine rise <50% from baseline and non-dialysis-dependent) vs negative outcome (creatinine rise >50% or dialysis dependence), assessed only among patients discharged alive (n=13 at ICU discharge, n=12 at hospital discharge, reflecting intercurrent deaths); Fisher’s exact test; ^2^Hierarchical composite clinical score analysed using Mann-Whitney U test; ICU, intensive care unit; IQR, interquartile range; CRRT, continuous renal replacement therapy.

No bleeding complications, citrate-related metabolic adverse events (e.g., citrate accumulation, symptomatic hypocalcaemia), heparin-induced thrombocytopenia, or other treatment-related adverse events were recorded during the study period.

### 3.4 Exploratory outcome: extracorporeal biomarker kinetics (pre- vs post-filter)

As an exploratory outcome, we compared paired pre- and post-filter biomarker concentrations on each occasion (1h and 24h) to evaluate whether the extracorporeal circuit removed or increased each biomarker, and whether this differed by anticoagulation strategy. CRP increased significantly from pre- to post-filter at both 1h (P <.001) and 24h (P = .018), with no evidence that this differed by anticoagulation strategy. MPO also increased significantly post-filter at 1h (P = .020), with a non-significant trend toward a larger increase in the heparin group (P = .077). GSH showed no significant overall pre-to-post change, but the direction and magnitude of this change differed significantly by anticoagulation strategy at 1h (P = .006). No significant pre-to-post or interaction effects were observed for GSSG, the GSSG/GSH ratio, or cfDNA on either occasion (**Table 4**, **Figure 4**).

**Table 4:** Pre- vs post-filter analysis by biomarker and occasion.

Domain	Biomarker	Occasion	n (obs/pts)	Position effect β [95% CI], P	Position × anticoagulation β [95% CI], P
Redox homeostasis					
	**GSH**	**T1**	**40/20**	**-4.45 [-16.49, 7.58], P = .448**	**35.61 [11.54, 59.67], P = .006**
	GSH	T2	32/16	-3.31 [-14.06, 7.45], P = .522	4.49 [-17.02, 26.00], P = .663
	GSSG	T1	38/20	-0.01 [-0.13, 0.12], P = .928	0.11 [-0.15, 0.37], P = .374
	GSSG	T2	32/16	0.00 [-0.06, 0.06], P = .903	0.02 [-0.10, 0.14], P = .751
	GSSG/GSH ratio	T1	38/20	0.75 [-0.25, 1.75], P = .126	-0.57 [-2.56, 1.43], P = .559
	GSSG/GSH ratio	T2	30/16	0.15 [-0.14, 0.43], P = .283	0.07 [-0.50, 0.64], P = .799
Neutrophil activation					
	**MPO**	**T1**	**38/19**	**14.53 [2.54, 26.52], P = .020**	**-21.35 [-45.33, 2.63], P = .077**
	MPO	T2	32/17	11.31 [-3.06, 25.67], P = .107	-21.72 [-50.37, 6.93], P = .122
Cellular injury					
	cfDNA	T1	38/19	-13.26 [-204.14, 177.61], P = .886	121.32 [-260.43, 503.07], P = .513
	cfDNA	T2	31/16	141.21 [-93.25, 375.66], P = .212	-382.73 [-851.64, 86.18], P = .096
Systemic inflammation					
	**CRP**	**T1**	**38/19**	**10.87 [6.29, 15.45], P<.001**	**-1.42 [-10.57, 7.73], P = .748**
	**CRP**	**T2**	**30/15**	**8.64 [1.64, 15.65], P = .018**	**-7.70 [-21.72, 6.31], P = .257**

Position effect = mean difference in measured concentration, Post-filter minus Pre-filter; positive values indicate a net increase and negative values a net decrease in measured concentration across the circuit. These differences should not be interpreted as direct evidence of biomarker generation, release, or removal without accounting for haemoconcentration, dilution, and flow-related factors (see Discussion). Position × Anticoagulation = difference in this Pre-Post change between Citrate and Heparin. Bold indicates P<.05. obs, observations; pts, patients; CI, confidence interval; GSH, reduced glutathione; GSSG, oxidized glutathione; MPO, myeloperoxidase; cfDNA, cell-free DNA; CRP, C-reactive protein.

**Figure 4:**
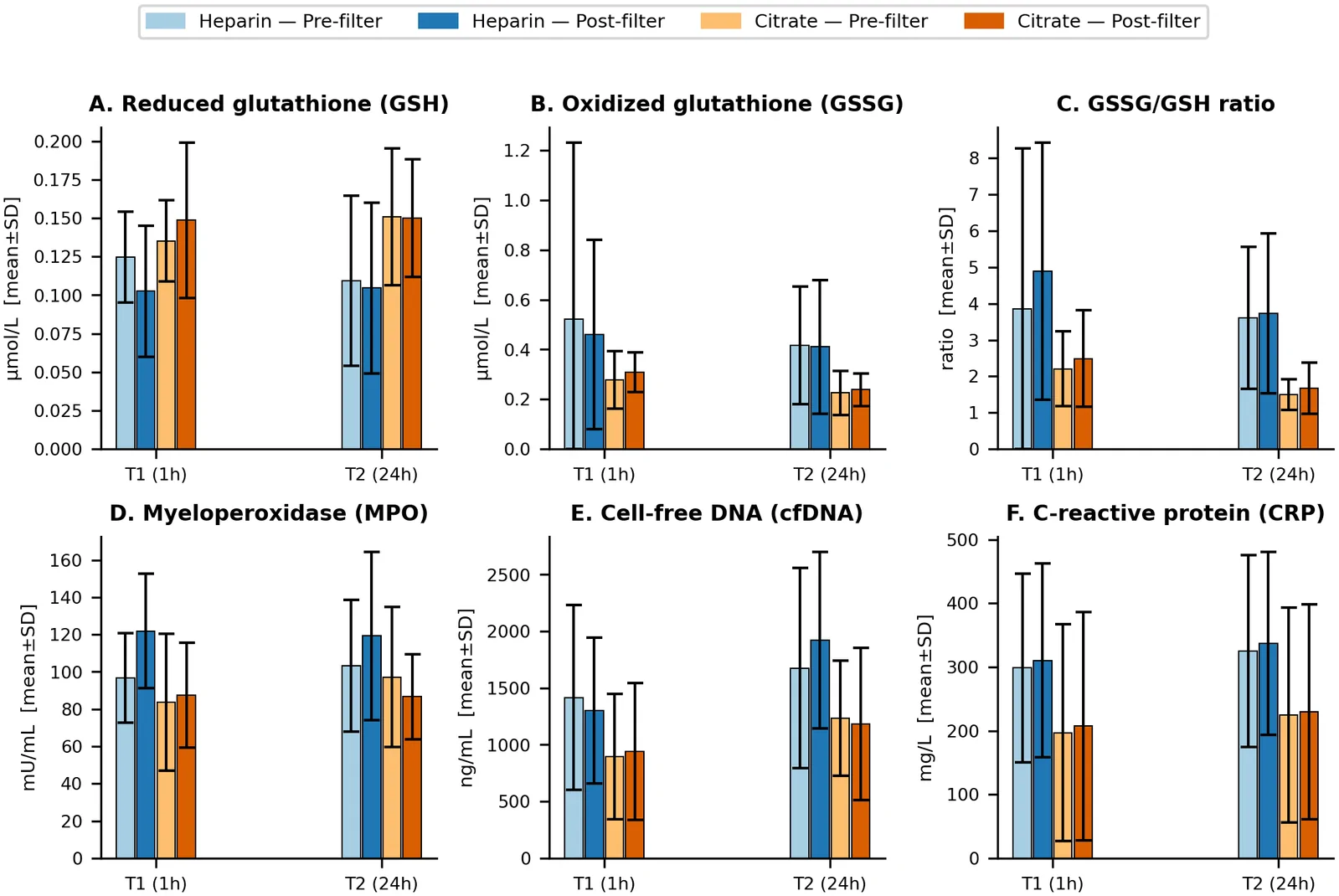
Pre- vs post-filter concentrations by anticoagulation strategy. Mean (± SD) pre-filter and post-filter concentrations of **(A)** GSH, **(B)** GSSG, **(C)** GSSG/GSH ratio, **(D)** MPO, **(E)** cfDNA and **(F)** CRP, by anticoagulation strategy, at 1h and 24h. Lower error bars are truncated at zero to reflect the biological non-negativity of measured concentrations; this affects only the visual display, not the underlying mean or SD. SD, standard deviation; GSH, reduced glutathione; GSSG, oxidized glutathione; MPO, myeloperoxidase; cfDNA, cell-free DNA; CRP, C-reactive protein.

## 4 Discussion

This study explored whether different anticoagulation strategies during CRRT are associated with distinct biological trajectories across complementary pathophysiological domains, viewing CRRT not only as renal support but also as a biological intervention. Rather than demonstrating a uniform biological effect of anticoagulation, our findings indicate that the biological response to CRRT is domain specific. GSH concentrations and circulating cfDNA, but not GSSG or the GSSG/GSH ratio, evolved significantly over time, whereas neutrophil activation exhibited a trajectory that differed according to the anticoagulation strategy. These observations suggest that the biological consequences of extracorporeal circulation are selective rather than homogeneous and support the concept that different pathophysiological domains respond differently to the extracorporeal environment.

This biological heterogeneity is consistent with current concepts of critical illness biology, including the unified theory of sepsis-associated acute kidney injury (8). MPO activity, a functional surrogate of neutrophil activation rather than a direct measure of MPO protein abundance since the assay quantifies enzymatic activity, showed the clearest association with anticoagulation strategy of the six biomarkers, despite neutrophil activation having been specified as a secondary rather than primary biological objective in the original protocol. A baseline-adjusted analysis at individual follow-up timepoints did not confirm this finding, illustrating how inference in this small pilot cohort depended on the analytical framework applied. The remaining biomarker comparisons should accordingly be weighted as exploratory throughout. Our findings regarding MPO within the neutrophil activation domain are consistent with previous studies of biomarker changes during citrate anticoagulation (17–19). Two aspects of this finding may be biologically informative and merit hypothesis-driven exploration in future, adequately powered, studies. First, the treatment-by-time coefficient for MPO was larger at 24h than at 1h (β=49.46 vs 33.14, **Table 2**), suggesting a cumulative rather than acute pattern of divergence between anticoagulation strategies, more consistent with progressive neutrophil activation over the course of CRRT than with an immediate contact-phase effect of the anticoagulant itself. Second, that the clearest signal emerged in neutrophil activation rather than in the redox domain, despite the latter being the protocol’s originally prioritized biological objective, may point toward a predominantly cellular/inflammatory, rather than purely oxidative, mechanism underlying anticoagulation-related biological modulation during CRRT, a distinction with potential mechanistic and therapeutic relevance that this pilot study is not designed to resolve. Because MPO reflects both neutrophil activation and oxidative biology, it may represent a biological bridge between innate immune activation and redox imbalance, illustrating that these domains should not be interpreted as biologically independent. Within the redox domain, the GSSG/GSH ratio remains the most informative indicator of systemic redox homeostasis and showed a treatment effect approaching, but not reaching, conventional statistical significance (P = .067). In a baseline-adjusted analysis extending the approach requested for MPO (**Supplementary Table 6**) to all six biomarkers, the GSSG/GSH ratio showed a significant between-group difference at 24h (β=−1.11, 95% CI [−1.99, −0.24], P = .017), with GSSG itself approaching significance at the same timepoint (P = .070); no other biomarker, including MPO, reached significance under this baseline-adjusted approach. Taken together with the longitudinal model, these results suggest that more than one biological domain may carry an exploratory signal in this pilot cohort, depending on the analytic approach applied, reinforcing our decision to treat all six biomarkers as exploratory and hypothesis-generating rather than to prioritize any single domain as confirmed.

Analysis of paired pre- and post-filter concentrations provided additional descriptive insight, which we interpret cautiously. CRP increased consistently across the extracorporeal circuit at both 1h and 24h, irrespective of anticoagulation strategy. Because CRP cannot be synthesized within the extracorporeal circuit, this pattern is compatible with haemoconcentration secondary to plasma water removal upstream of the post-filter sampling site, non-selective release of CRP previously retained within the membrane’s secondary protein layer through competitive protein displacement (20–23), or a combination of both mechanisms.

A companion analysis of this cohort investigating biomarker mass transfer across the circuit showed that CRP was the only biomarker detectable in the ultrafiltrate/effluent (24, 25), whereas GSH, GSSG, MPO and cfDNA were undetectable at all sampling points. This finding supports, but does not prove, selective trans-membrane transport of CRP. For the remaining biomarkers, the absence of effluent detection may reflect either negligible membrane transport or concentrations below the analytical detection limit after dilution within the effluent stream. The latter explanation is biologically more plausible for low-abundance analytes such as GSSG than for GSH, whose substantially higher circulating concentration would be expected to generate detectable effluent concentrations even with modest membrane clearance. For MPO, an additional methodological consideration applies, as the assay quantifies enzymatic activity rather than MPO protein mass; consequently, a negative effluent result cannot distinguish between absence of MPO protein and loss of detectable enzymatic activity under effluent conditions.

The observed biomarker pattern is not entirely consistent with a uniform haemoconcentration effect. Whereas CRP showed a consistent post-filter increase and MPO a smaller but measurable change, GSSG, the GSSG/GSH ratio and cfDNA remained essentially unchanged across the circuit. Such marker-specific behaviour is more compatible with selective biological interactions at the blood–membrane interface than with non-specific concentration of retained plasma constituents alone. Although paired total protein measurements were not available to test the haemoconcentration hypothesis directly, the magnitude of the expected effect can be bounded from the prescribed flows: the estimated filtration fraction was 4.5% (median; IQR 1.2–5.8), corresponding to a post-filter haemoconcentration factor of approximately 1.05. Uniform haemoconcentration would therefore be expected to raise all measured analytes by a similar and small proportion, which is not the pattern observed. Haematocrit was an estimated value in 12 of the 20 patients, but recalculating under assumptions of 0.25 and 0.35 shifts the median filtration fraction only from 4.4% to 4.7%. Because these estimates derive from prescribed rather than directly measured flows, they are offered as an order-of-magnitude benchmark and the resulting inferences should be regarded as exploratory.

The corresponding effect at the opposite end of the circuit acts in the reverse direction: because regional citrate was infused upstream of the pre-filter sampling site used for the longitudinal analyses, we quantified its expected haemodilution effect using the prescribed blood flow and citrate infusion rates. The estimated dilution (3.0–3.9%; mean 3.6%) was small and highly consistent across patients. Correcting citrate-group MPO concentrations for this effect did not materially alter the time × anticoagulation interaction (P = .006 versus P = .008 uncorrected), indicating that citrate-induced haemodilution is unlikely to explain the principal finding. Moreover, the significant pre-to-post-filter change in GSH observed at 1h suggests that at least part of the biological modulation associated with anticoagulation strategy may originate within the extracorporeal circuit itself rather than reflecting systemic biology alone. Dilution at the pre-filter port (≈3.6%) and concentration at the post-filter port (≈5%) are thus of comparable magnitude and act in the same direction, both tending to inflate any apparent increase across the filter.

Taken together, these findings support the concept that CRRT is not biologically neutral but may interact selectively with distinct pathophysiological domains, both systemically and at the blood–extracorporeal interface. Although these exploratory observations require confirmation in larger studies, they suggest that biological responses to CRRT may be domain-specific and therefore unlikely to translate uniformly into clinical outcomes. Future investigations should combine biological subphenotyping, multi-omic profiling and direct measurements of haemoconcentration markers to further advance precision extracorporeal medicine.

### 4.1 Limitations

This pilot study has several limitations. First, the small sample size (n=20) substantially limited statistical power for both biological and clinical comparisons. Second, recruitment was markedly disrupted by the COVID-19 pandemic, during which 103 otherwise eligible patients could not be enrolled (**Figure 2**), extending recruitment over almost four years and potentially increasing temporal heterogeneity in clinical practice. Third, the study was not designed or powered to evaluate clinical outcomes or biological subphenotypes. Fourth, actual delivered CRRT dose and filter lifespan were not prespecified study objectives and were therefore not systematically recorded. Fifth, the healthy reference cohort comprised eight participants, fewer than the 22 provided for in the original protocol; the resulting values should therefore be read as indicative rather than as established normative ranges, although no comparison reported here is powered on that cohort. Finally, paired total protein measurements were unavailable at each sampling timepoint, precluding direct quantification of haemoconcentration across the extracorporeal circuit, although its expected magnitude could be bounded from the prescribed flows (see Discussion).

Several analytical limitations should also be considered. First, because recruitment extended over almost four years owing to the COVID-19 pandemic, storage duration before biomarker analysis varied substantially. Samples were stored for 11–1034 days before GSH/GSSG analysis, 97–1473 days before MPO analysis, and 432–1808 days before cfDNA analysis. Importantly, storage duration did not differ significantly between anticoagulation groups for any biomarker, minimizing the likelihood of differential bias. Nevertheless, prolonged storage may have contributed to measurement variability, particularly for the redox-sensitive analytes GSH and GSSG (26), for which some degree of degradation cannot be completely excluded. Second, inter-batch quality-control metrics were unavailable for GSH, GSSG and cfDNA because these analyses were performed by an external university laboratory that reported validated final concentrations without retaining calibration-curve or replicate-control data. Finally, MPO was analyzed in a single analytical session, precluding formal assessment of inter-batch reproducibility, and replicate-based intra-assay coefficients of variation were not available. However, review of the archived raw analytical output confirmed highly linear kinetic responses across all seven calibration standards (R² 0.9974–0.9994) and an excellent fit of the seven-point cubic calibration curve (R²=0.99989; Section 2.6.2).

The assessment of clinical outcomes is additionally constrained by competing mortality, as renal recovery could only be evaluated among survivors, reducing the effective sample size for these analyses. Furthermore, no formal correction for multiple comparisons was applied across the six biomarkers. Consequently, all biomarker analyses, including MPO, should be interpreted as exploratory and hypothesis-generating. Although the longitudinal MPO signal remained stable across outlier-exclusion and citrate-dilution sensitivity analyses, it was not confirmed in baseline-adjusted analyses at individual follow-up timepoints, reinforcing the need for confirmation in adequately powered prospective studies. For two biomarkers the exclusion criterion was not inconsequential: the GSH time effect and the GSSG/GSH ratio treatment effect were attenuated, though not reversed, when outlier observations were retained (**Supplementary Table 3**).

Rather than representing only a limitation, however, the present pilot study also provides information that may guide future research. Using the observed effect sizes and standard errors, we estimated the sample size required to confirm biomarker interactions of the magnitude observed here with 80% and 90% power (two-sided α=0.05), extending the *a priori* MPO calculation based on Bos et al. (17) to all biomarkers (**Table 5**). GSH and cfDNA would require prohibitively large sample sizes (n>600–1400) to confirm the modest, non-significant interactions observed here, suggesting that any true anticoagulation-related effect on these markers, if present, is likely too small to be clinically pursued with this design. In contrast, GSSG, the GSSG/GSH ratio, and CRP at 1h showed projected sample size requirements (approximately 85–470 patients) that would be achievable in a multicentre study, making them reasonable candidates for a confirmatory trial.

**Table 5:** Projected sample size required to detect an interaction effect for each biomarker.

Biomarker	Contrast	Current n (pts)	│β│ observed	n required (80% power)	n required (90% power)
Reduced glutathione (GSH)	T1 vs T0	20	7.87	820	1098
Reduced glutathione (GSH)	T2 vs T0	20	8.14	893	1195
Oxidized glutathione (GSSG)	T1 vs T0	19	0.34	83	112
Oxidized glutathione (GSSG)	T2 vs T0	19	0.23	203	271
GSSG/GSH ratio	T1 vs T0	19	1.50	137	183
GSSG/GSH ratio	T2 vs T0	19	1.41	177	236
Cell-free DNA (cfDNA)	T1 vs T0	20	114.50	589	788
Cell-free DNA (cfDNA)	T2 vs T0	20	94.00	1093	1464
C-reactive protein (CRP)	T1 vs T0	20	25.61	350	468
C-reactive protein (CRP)	T2 vs T0	20	9.19	3406	4560

Projected sample size required to detect, with adequate power, an interaction effect of the same magnitude as observed in this pilot study for each biomarker (MPO omitted, as it was the only biomarker for which a literature-based power calculation was incorporated during the study (see Methods 2.9)). Projections are derived from the observed effect estimate and its standard error and should be interpreted as first-order estimates: pilot-derived effect sizes are inherently imprecise, and these figures can vary substantially depending on which contrast (1h vs 24h) is used, as illustrated above. They are intended to inform, not guarantee, the design of future confirmatory studies. pts, patients; β, fixed-effect coefficient, in each biomarker’s original units (not standardized); MPO, myeloperoxidase.

The absence of consistent differences in previous trials may reflect, at least in part, the complexity of translating selective biological modulation into measurable clinical benefit. Randomized trials directly comparing RCA and systemic heparin anticoagulation during CRRT have progressively increased in size from single-centre studies of fewer than 50 patients (27) to large multicentre trials (28, 29), including the RICH trial (n = 596) (29), yet none has demonstrated a mortality difference between strategies. A meta-analysis (30) pooling 14 trials (n = 1134), supported by trial sequential analysis, likewise found no difference in mortality (RR 0.97, 95% CI 0.84–1.13). Subsequent reviews and meta-analyses (31, 32) yielded similar findings, with no statistically significant differences in ICU length of stay, all-cause mortality, duration of CRRT, renal recovery, or adverse events across most evaluated anticoagulation strategies. A notable exception was the earlier trial by Oudemans-van Straaten (33), involving 200 critically ill patients, which reported a survival benefit in the citrate-randomized group. This *post hoc* effect was particularly apparent in surgical patients and in those with sepsis or severe multiple organ failure, raising the possibility that citrate may modulate systemic inflammation. Taken together, these data suggest that the absence of an overall mortality effect is unlikely to be explained simply by inadequate sample size.

Beyond their statistical implications, the sample-size projections presented in **Table 5** may also have methodological implications for future research. Most randomized trials comparing regional citrate and systemic heparin have primarily focused on patient-centered outcomes such as mortality, renal recovery or filter lifespan (30–32). However, these distal outcomes represent the integrated consequence of multiple interacting biological pathways. If different anticoagulation strategies exert domain-specific rather than uniform biological modulation, as suggested by this pilot study, expecting consistent improvements in clinical outcomes before establishing reproducible effects across the relevant biological domains may be premature. A more translational research strategy may therefore consist of progressively linking biological modulation to clinically meaningful outcomes: first identifying which pathophysiological domains are consistently modified, then determining the biological subphenotypes in which these changes are most relevant and finally evaluating whether such targeted modulation translates into improved clinical outcomes. From this perspective, the sample-size estimates derived from the present pilot study should not be interpreted solely as planning tools for future trials but also as a means of prioritizing the biological domains most likely to justify confirmatory investigation.

This framework should not, however, be read as implying that previous trials were simply underpowered: the trial sequential analysis noted above (30) suggests that the cumulative sample size already achieved may be sufficient to exclude a clinically important mortality effect. Rather, as with many surrogate markers in critical care, a reproducible, adequately powered biological effect, such as the domain-specific modulation of neutrophil activation observed here, need not translate into a measurable difference in a distal, multiply determined outcome such as mortality, particularly if the effect is heterogeneous across patients or attenuated by compensatory physiological mechanisms. Whether a biological effect exists and whether it is clinically meaningful therefore remain distinct questions, and the absence of a mortality signal across existing trials should not be taken as evidence against domain-specific biological modulation. Accordingly, the implications of these findings for precision extracorporeal medicine should be regarded as hypothesis-generating and require validation in larger multicentre studies. Ultimately, defining reproducible biological effects across pathophysiological domains may represent a necessary step towards precision extracorporeal medicine.

## 5 Conclusions

In conclusion, our findings suggest that different anticoagulation strategies during CRRT may be associated with domain-specific biological modulation rather than a uniform systemic response, evident both in systemic biomarker trajectories and in biomarker kinetics across the extracorporeal circuit. Understanding these distinct pathophysiological trajectories may, if confirmed, eventually contribute to the identification of biologically relevant subphenotypes. However, given this study’s small sample size, its exploratory design, and the fact that our principal finding was not confirmed in a baseline-adjusted sensitivity analysis, these implications remain hypothetical. Future, adequately powered multicentre studies with prospectively defined biological endpoints are warranted to determine whether domain-specific biological signals of this kind are reproducible and clinically meaningful.

## Data Availability

The raw data supporting the conclusions of this article will be made available by the authors, without undue reservation.
