# Supplementary material for: Beyond renal support: biological modulation across pathophysiological domains during continuous renal replacement therapy — a randomized pilot study
Source: Front Nephrol. 2026 Sep 18;6:1932419. doi: 10.3389/fneph.2026.1932419 (PMC13643268; doi:10.3389/fneph.2026.1932419)
Supplement: Supplementary file 1 [file DataSheet1.pdf]

Supplementary Material

1 Supplementary Table S1. Outlier exclusion (|z| > 2 SD)

Biomarker	Timepoint	Case excluded	Group	z-score
GSH	T2 (24h)	Case 29	Heparin	−2.33
GSSG/GSH ratio	T0	Case 1	Citrate	2.20
MPO	T2 (24h)	Case 27	Heparin	2.10
MPO	T2 (24h)	Case 30	Citrate	2.25
cfDNA	T0	Case 4	Heparin	2.44
cfDNA	T1 (1h)	Case 4	Heparin	2.46
cfDNA	T2 (24h)	Case 27	Heparin	2.21
CRP	T0	Case 29	Heparin	2.06
CRP	T2 (24h)	Case 28	Citrate	2.01

GSSG showed no observations exceeding this threshold. Exclusion criterion defined by the study team prior to data analysis (see Methods 2.9).

2 Supplementary Table S2. Missing sample summary, by biomarker

Biomarker	Possible (n)	Collected	Not collected	Outlier-excluded	Analysed
GSH	60	57	3	1	56
GSSG	60	54	6	0	54
GSSG/GSH ratio	60	52	8	1	51
MPO	60	55	5	2	53
cfDNA	60	56	4	3	53
CRP	60	55	5	2	53

Possible = 20 patients × 3 timepoints. Missingness was concentrated at the 24-hour timepoint across all six biomarkers, consistent with clinically plausible causes (ICU discharge, mortality, or CRRT discontinuation by 24h).

3 Supplementary Table S3. Sensitivity analysis: with and without outlier exclusion, all biomarkers

Comparison	Exclusion	N (obs/pts)	Time effect, P	Treatment effect, P	Time x Treatment
GSH	Excluded (primary)	56/20	.011	.150	.683
	Retained (sensitivity)	57/20	.138	.052	.356
GSSG	Excluded (primary)	54/19	.570	.553	.361
	Retained (sensitivity)	54/19	.570	.553	.361
GSSG/GSH ratio	Excluded (primary)	51/19	.543	.067	.482
	Retained (sensitivity)	52/19	.450	.385	.155
**MPO**	**Excluded (primary)**	**53/19**	**.209**	**.054**	**.008**
	**Retained (sensitivity)**	**55/19**	**.209**	**.089**	**.015**
cfDNA	Excluded (primary)	53/20	.008	.190	.664
	Retained (sensitivity)	56/20	.009	.122	.717
CRP	Excluded (primary)	53/20	.252	.051	.772
	Retained (sensitivity)	55/20	.319	.055	.492

Myeloperoxidase (bold) is the only biomarker for which the time × treatment interaction is significant under both approaches. Time and Treatment effect P-values in this table are derived from likelihood-ratio χ^2 tests and are not directly comparable to the omnibus F-test P-values for these same main effects reported in manuscript Table 2; the Time × Treatment interaction for MPO, the primary inferential target, is numerically concordant between both approaches (P=.008 in each); this concordance does not necessarily extend to the other five biomarkers, where Time × Treatment P-values also differ numerically between the two approaches, consistent with the pattern seen for the main effects.

4 Methods

As noted in the main text (Section 2.9, Statistical analysis), the primary longitudinal analyses were performed using linear mixed-effects models with fixed effects for time, anticoagulation strategy and their interaction, and patient identity as a random intercept. To complement these models, this Supplementary Material provides pairwise comparisons between individual sampling time points for each biomarker.

Pairwise time-point comparisons were performed using the paired Student's t-test for normally distributed variables and the Wilcoxon signed-rank test otherwise, according to the results of the Shapiro–Wilk normality test. These pairwise analyses are exploratory, are not adjusted for multiple comparisons, and should be interpreted as hypothesis-generating; they are reported here to complement, not replace, the primary mixed-model analyses reported in Table 2 and Table 4 of the main manuscript. Time points are abbreviated as follows: T0, before CRRT initiation; T1 pre/post, one hour after initiation, pre-filter/post-filter; T2 pre/post, twenty-four hours after initiation, pre-filter/post-filter.

Supplementary Table S4. Pairwise comparisons between individual timepoints, by anticoagulation group

Biomarker	Group	Contrast	n (pairs)	P
GSH	**Heparin**	**T0 vs T1**	**10**	**.010**
	Heparin	T1 vs T2	8	.844
	Heparin	T0 vs T2	8	.109
	Citrate	T0 vs T1	10	.193
	Citrate	T1 vs T2	8	.312
	Citrate	T0 vs T2	8	.195
GSSG	Heparin	T0 vs T1	10	.375
	Heparin	T1 vs T2	8	.945
	Heparin	T0 vs T2	8	.641
	Citrate	T0 vs T1	9	.426
	Citrate	T1 vs T2	8	.547
	Citrate	T0 vs T2	8	.383
GSSG/GSH ratio	Heparin	T0 vs T1	9	.570
	Heparin	T1 vs T2	7	.469
	Heparin	T0 vs T2	6	.312
	Citrate	T0 vs T1	8	.078
	Citrate	T1 vs T2	8	.078
	**Citrate**	**T0 vs T2**	**8**	**.039**
**MPO**	**Heparin**	**T0 vs T1**	**10**	**.010**
	Heparin	T1 vs T2	8	.945
	**Heparin**	**T0 vs T2**	**8**	**.023**
	Citrate	T0 vs T1	9	.496
	Citrate	T1 vs T2	7	.688
	Citrate	T0 vs T2	7	.156
cfDNA	Heparin	T0 vs T1	9	.426
	Heparin	T1 vs T2	6	.438
	Heparin	**T0 vs T2**	**6**	**.031**
	Citrate	T0 vs T1	10	.193
	Citrate	T1 vs T2	8	.148
	Citrate	T0 vs T2	8	.148
CRP	Heparin	T0 vs T1	9	1.000
	Heparin	T1 vs T2	7	.156
	Heparin	T0 vs T2	7	.578
	Citrate	T0 vs T1	10	.375
	Citrate	T1 vs T2	7	.375
	Citrate	T0 vs T2	7	.469

Bold indicates P<.05. Given the exploratory nature of these pairwise comparisons and the absence of a multiplicity correction, results should be interpreted descriptively alongside the primary longitudinal model (Table 2).

Supplementary Table S5.1. Pairwise comparisons of pre-filter/post-filter GSH concentrations between individual time points (whole cohort).

Comparison	n	Mean difference (SD)	Test statistic	P
T0 vs T1 pre-filter	20	-19 (25)	t(19) = -3.326	.004*
T0 vs T1 post-filter	20	-14 (39)	t(19) = -1.666	.112
T1 pre- vs post-filter	20	4 (32)	t(19) = 0.615	.546
T0 vs T2 pre-filter	17	-22 (66)	t(16) = -1.352	.195
T0 vs T2 post-filter	17	-19 (60)	t(16) = -1.275	.220
T2 pre- vs post-filter	17	3 (21)	t(16) = 0.581	.569

*GSH in µmol/L, mean difference (SD). Paired Student's t-test. *P < 0.05. Concentrations are reported in µmol/L; the raw assay output (µmol/mL) was converted as described in Methods. The number of complete pairs differs between contrasts: n=20 for comparisons involving only T0 and the 1-hour timepoint (rows 1–3) and n=17 for those involving the 24-hour timepoint (rows 4–6), owing to missing 24-hour samples.*

Supplementary Table S5.2. Pairwise comparisons of pre-filter/post-filter GSSG concentrations between individual time points (whole cohort).

Comparison	n	Median (P25–P75) time 1	Median (P25–P75) time 2	Wilcoxon W	P
T0 vs T1 pre-filter	19	0.290 (0.200–0.492)	0.317 (0.213–0.381)	89.0	.829
T0 vs T1 post-filter	19	0.290 (0.200–0.492)	0.361 (0.222–0.500)	63.0	.347
T1 pre- vs post-filter	19	0.317 (0.213–0.381)	0.361 (0.222–0.500)	64.0	.369
T0 vs T2 pre-filter	16	0.290 (0.200–0.492)	0.264 (0.195–0.373)	76.0	.706
T0 vs T2 post-filter	16	0.290 (0.200–0.492)	0.280 (0.196–0.339)	81.0	.528
T2 pre- vs post-filter	16	0.264 (0.195–0.373)	0.280 (0.196–0.339)	63.0	.821

GSSG in µmol/L, median (P25–P75). The raw assay output (nmol/mL) is numerically identical in these units. Wilcoxon signed-rank test. No pairwise comparison reached statistical significance. The number of complete pairs differs between contrasts: n=19 for comparisons involving only T0 and the 1-hour timepoint and n=16 for those involving the 24-hour timepoint

Supplementary Table S5.3. Pairwise comparisons of the pre-filter/post-filter GSSG/GSH ratio between individual time points (whole cohort).

Comparison	n	Median (P25–P75) time 1	Median (P25–P75) time 2	Wilcoxon W	P
T0 vs T1 pre-filter	18	2.334 (1.973–4.129)	2.226 (1.619–3.842)	118.0	.167
T0 vs T1 post-filter	18	2.334 (1.973–4.129)	2.825 (2.174–4.791)	54.0	.181
T1 pre- vs post-filter	18	2.226 (1.619–3.842)	2.825 (2.174–4.791)	43.0	.067
T0 vs T2 pre-filter	15	2.334 (1.973–4.129)	1.632 (1.235–3.094)	90.0	.017*
T0 vs T2 post-filter	15	2.334 (1.973–4.129)	1.663 (1.221–2.974)	89.0	.107
T2 pre- vs post-filter	14	1.632 (1.235–3.094)	1.663 (1.221–2.974)	42.0	.542

*GSSG/GSH ratio, median (P25–P75). The ratio is dimensionless and is presented ×1000 for legibility, computed from concentrations expressed in µmol/L. Wilcoxon signed-rank test. *P < 0.05. The number of complete pairs differs between contrasts: n=18 for comparisons involving only T0 and the 1-hour timepoint and n=14-15 for those involving the 24-hour timepoint (n=14 in comparison involving pre and post-filter 24-hour timepoint).*

Supplementary Table S5.4. Pairwise comparisons of pre-filter/post-filter MPO activity between individual time points (whole cohort).

Comparison	n	Mean difference (SD) / Median (IQR)	Test statistic	P
T0 vs T1 pre-filter	19	11.993 (31.097)	t(18) = 1.681	.110
T0 vs T1 post-filter	19	-3.095 (25.784)	t(18) = -0.523	.607
T1 pre- vs post-filter	19	-15.088 (28.099)	t(18) = -2.341	.031*
T0 vs T2 pre-filter	17	96.823 (76.424) vs 88.554 (37.345)	W = 75	.963
T0 vs T2 post-filter	17	-0.776 (31.581)	t(16) = -0.101	.921
T2 pre- vs post-filter	17	88.554 (37.345) vs 98.272 (63.297)	W = 75	.963

*MPO activity in mU/mL. Rows 1–4: paired Student's t-test, mean difference (SD). Rows 5–6: Wilcoxon signed-rank test (W statistic), median (IQR) at each timepoint, used given non-normality of the T2 distributions. *P < 0.05. The number of complete pairs differs between contrasts: n=19 for comparisons involving only T0 and the 1-hour timepoint and n=17 for those involving the 24-hour timepoint*

Supplementary Table S5.5. Pairwise comparisons of pre-filter/post-filter cfDNA concentrations between individual time points (whole cohort).

Comparison	n	Mean difference (SD)	Test statistic	P
T0 vs T1 pre-filter	20	-104.877 (367.291)	t(19) = -1.277	.217
T0 vs T1 post-filter	20	-69.712 (244.197)	t(19) = -1.277	.217
T1 pre- vs post-filter	20	35.165 (420.767)	t(19) = 0.374	.713
T0 vs T2 pre-filter	16	-376.987 (522.665)	t(15) = -2.885	.011*
T0 vs T2 post-filter	16	-474.757 (461.600)	t(15) = -4.114	< .001**
T2 pre- vs post-filter	16	-97.8 (466.83)	t(15) = -0.838	.415

*cfDNA in ng/mL, mean difference (SD). Paired Student's t-test. *P < 0.05, **P < 0.01. The number of complete pairs differs between contrasts: n=20 for comparisons involving T0 and 1-hour timepoint (rows 1-3) and n=16 for those involving the 24-hour timepoint (rows 4-6), owing to missing 24-hour samples.*

Supplementary Table S5.6. Pairwise comparisons of pre-filter/post-filter CRP concentrations between individual time points (whole cohort).

Comparison	n	Mean difference (SD)	Test statistic	P
T0 vs T1 pre-filter	19	-3.17 (66.94)	t(18) = -0.206	.839
T0 vs T1 post-filter	20	-20.95 (70.62)	t(19) = -1.327	.200
T1 pre- vs post-filter	19	-10.83 (9.90)	t(18) = -4.768	< .001**
T0 vs T2 pre-filter	16	-28.20 (114.38)	t(15) = -0.986	.340
T0 vs T2 post-filter	16	-36.77 (115.28)	t(15) = -1.276	.221
T2 pre- vs post-filter	16	-8.57 (13.49)	t(15) = -2.540	.023*

*CRP in mg/L, mean difference (SD), calculated as the earlier minus the later measurement, so that negative values denote an increase. Paired Student's t-test. *P < 0.05, **P < 0.01. The number of complete pairs differs between contrasts (n=19–20 for comparisons involving only T0 and the 1-hour timepoint, n=16 for those involving the 24-hour timepoint).*

5 Supplementary Table S6. Baseline-adjusted analysis (ANCOVA) for all biomarkers

Linear regression of follow-up concentration on treatment group and baseline (T0) concentration as covariate, using all available observations with outlier exclusion (|z|>2 SD) as in the primary analysis. β represents the adjusted between-group difference (citrate minus heparin) at each timepoint, in each biomarker's original units.

Biomarker	Timepoint	n	β (Citrate−Heparin)	95% CI	P
GSH, µmol/L	T1	20	0.70	[-16.90, 18.31]	.934
	T2	16	23.77	[-24.85, 72.40]	.310
GSSG, µmol/L	T1	19	-0.24	[-0.74, 0.25]	.314
	T2	16	-0.15	[-0.32, 0.01]	.070
GSSG/GSH ratio	T1	17	-1.95	[-5.42, 1.52]	.247
	**T2**	**14**	**-1.11**	**[-1.99, -0.24]**	**.017**
MPO, mU/mL	T1	19	14.42	[-13.71, 42.55]	.293
	T2	15	8.36	[-19.47, 36.19]	.525
cfDNA, ng/mL	T1	19	-149.26	[-532.39, 233.87]	.421
	T2	14	35.19	[-585.67, 656.06]	.903
CRP, mg/L	T1	19	13.27	[-58.14, 84.68]	.699
	T2	14	-62.61	[-181.34, 56.12]	.270

Bold indicates P<.05. This analysis extends the baseline-adjusted comparison to all six biomarkers, for completeness and methodological consistency. The GSSG/GSH ratio showed a significant baseline-adjusted between-group difference at T2 (β=−1.11, 95% CI [−1.99, −0.24], P=.017); GSSG itself approached significance at the same timepoint (P=.070). No other biomarker, including MPO, reached significance in this baseline-adjusted analysis. These findings should be interpreted as hypothesis-generating and are not corrected for multiplicity, consistent with the exploratory framework applied throughout this study.

6 Supplementary Table S7. Hierarchical composite clinical score, by patient

Per-patient derivation of the exploratory composite clinical score (0–4) described in Methods 2.9 and reported in Results 3.3. CRRT duration cutoffs: overall study mean (3.0 days) for the early/late mortality distinction (Score 0/1); overall study median (2.5 days) for the short/prolonged CRRT distinction among survivors with positive renal recovery (Score 3/4).

Case	Group	Hospital status	CRRT duration (days)	Renal function at discharge	Composite score
1	Citrate	Deceased	1	Deceased	0
2	Heparin	Survived	1	Normal	4
3	Citrate	Deceased	1	Deceased	0
4	Heparin	Survived	2	Cr rise >50% / dialysis	2
5	Heparin	Deceased	9	Deceased	1
6	Heparin	Deceased	4	Deceased	1
7	Citrate	Survived	3	Normal	3
21	Citrate	Deceased	1	Deceased	0
22	Heparin	Survived	4	Normal	3
23	Citrate	Survived	3	Normal	3
24	Heparin	Survived	1	Normal	4
25	Heparin	Survived	3	Normal	3
26	Citrate	Deceased	8	Deceased	1
27	Heparin	Deceased	3	Deceased	0
28	Citrate	Survived	2	Normal	4
29	Heparin	Survived	5	Normal	3
30	Citrate	Survived	2	Normal	4
31	Citrate	Survived	1	Normal	4
32	Heparin	Deceased	5	Deceased	1
33	Citrate	Survived	1	Normal	4

Distribution compared between anticoagulation groups using the Mann-Whitney U test (U=47.0, P=.846).

7 Note

Underlying patient-level data supporting these supplementary analyses are available from the corresponding author on reasonable request, consistent with the Data Availability Statement in the main manuscript.
