# Supplementary material for: Beyond renal support: biological modulation across pathophysiological domains during continuous renal replacement therapy — a randomized pilot study
Source: Front Nephrol. 2026 Sep 18;6:1932419. doi: 10.3389/fneph.2026.1932419 (PMC13643268; doi:10.3389/fneph.2026.1932419)
Supplement: Supplementary file 2 [file DataSheet2.pdf]

CONSORT 2010 Checklist

Beyond Renal Support: Biological Modulation Across Pathophysiological Domains During Continuous Renal Replacement Therapy — A Randomized Pilot Study

Completed according to the CONSORT 2010 Statement (Schulz KF, Altman DG, Moher D; CONSORT Group. CONSORT 2010 Statement: updated guidelines for reporting parallel group randomised trials. BMJ. 2010;340:c332).

Section/topic	Item No	Checklist item	Reported on page/section	Status
**Title and abstract**				
	1a	Identification as a randomised trial in the title	Title ("...A Randomized Pilot Study")	Reported
	1b	Structured summary of trial design, methods, results, and conclusions	Abstract	Reported
**Introduction**				
	2a	Scientific background and explanation of rationale	Introduction (Section 1)	Reported
	2b	Specific objectives or hypotheses	Introduction, final paragraph	Reported
**Methods**				
	3a	Description of trial design, including allocation ratio	2.1 Study design	Reported
	3b	Important changes to methods after trial commencement, with reasons	2.2 Ethics statement<br>2.5 Sample collection	Reported
	4a	Eligibility criteria for participants	2.3 Study population	Reported
	4b	Settings and locations where data were collected	2.1 Study design (bicentric ICUs)	Reported
	5	Interventions for each group with sufficient detail for replication	2.4 CRRT and anticoagulation protocol	Reported
	6a	Completely defined pre-specified primary and secondary outcomes	2.7 Outcomes	Reported
	6b	Any changes to trial outcomes after the trial commenced, with reasons	–	Not applicable (no changes)
	7a	How sample size was determined	2.9 Statistical analysis	Reported
	7b	Explanation of any interim analyses and stopping guidelines	–	Not applicable (no interim analyses or prespecified stopping guidelines)
	8a	Method used to generate the random allocation sequence	2.1 Study design	Reported
	8b	Type of randomisation; details of restriction (e.g. blocking, stratification)	2.1 Study design (permuted blocks of 4)	Reported
	9	Mechanism used to implement the random allocation sequence, and allocation concealment steps	2.1 Study design (sealed opaque envelopes)	Reported

Section/topic	Item No	Checklist item	Reported on page/section	Status
	10	Who generated the allocation sequence, who enrolled participants, who assigned participants to interventions	2.1 Study design	Reported
	11a	If done, who was blinded after assignment, and how	2.6 Biomarker analysis	Reported
	11b	If relevant, description of the similarity of interventions	–	Not applicable (open-label)
	12a	Statistical methods used to compare groups for primary and secondary outcomes	2.9 Statistical analysis	Reported
	12b	Methods for additional analyses, such as subgroup analyses and adjusted analyses	2.9 Statistical analysis	Reported
**Results**				
	13a	For each group, the numbers of participants randomly assigned, receiving intended treatment, and analysed for the primary outcome	3.1 Patient enrolment and baseline characteristics; Figure 2	Reported
	13b	For each group, losses and exclusions after randomisation, with reasons	Figure 2 (patient flow diagram)	Reported
	14a	Dates defining the periods of recruitment and follow-up	3.1 Patient enrolment (June 2018–May 2022)	Reported
	14b	Why the trial ended or was stopped	Limitations (COVID-19 impact on recruitment)	Reported
	15	A table showing baseline demographic and clinical characteristics for each group	Table 1	Reported
	16	For each group, number of participants included in each analysis and whether the analysis was by original assigned groups	3.1–3.4; Tables 2–4	Reported
	17a	For each primary and secondary outcome, results for each group and the estimated effect size and its precision	3.2–3.4; Tables 2–4	Reported
	17b	For binary outcomes, presentation of both absolute and relative effect sizes	3.3 Secondary outcomes (Table 3)	Partially reported (event/counts proportions reported; relative effect estimates not provided)
	18	Results of any other analyses performed, including subgroup and adjusted analyses	3.4 Exploratory outcome (pre- vs post-filter kinetics)	Reported
	19	All important harms or unintended effects in each group	3.3 Secondary outcome: clinical outcomes	Reported
**Discussion**				
	20	Trial limitations, addressing sources of potential bias, imprecision, and multiplicity of analyses	4.1 Limitations	Reported
	21	Generalisability of the trial findings	Discussion / 4.1 Limitations	Reported
	22	Interpretation consistent with results, balancing benefits and harms, and considering other evidence	Discussion	Reported
**Other information**				

Section/topic	Item No	Checklist item	Reported on page/section	Status
	23	Registration number and name of trial registry	Abstract; 2.2 Ethics statement (EudraCT, ClinicalTrials.gov)	Reported
	24	Where the full trial protocol can be accessed, if available	Available from the corresponding author on reasonable request	Available on request
	25	Sources of funding and other support, and role of funders	Funding section	Reported

Note: This checklist is provided as supplementary material to support editorial and peer review, consistent with journal recommendations for reporting of randomised trials.
