# Supplementary material for: Beyond renal support: biological modulation across pathophysiological domains during continuous renal replacement therapy — a randomized pilot study
Source: Front Nephrol. 2026 Sep 18;6:1932419. doi: 10.3389/fneph.2026.1932419 (PMC13643268; doi:10.3389/fneph.2026.1932419)
Supplement: Supplementary file 3 [file SupplementaryFile1.pdf]

Supplementary Methods

Anticoagulation protocols

1. Systemic unfractionated heparin anticoagulation

In patients allocated to systemic unfractionated heparin (UFH), anticoagulation was initiated with a loading dose of 2,000-5,000 IU administered into the extracorporeal circuit, followed by a continuous infusion of 5-10 IU/kg/h. The infusion rate was subsequently adjusted according to activated partial thromboplastin time (aPTT), with a target range of 35-45 s (up to approximately 1.4 times control). aPTT was measured at least once daily, and dose adjustments also considered the individual bleeding/thrombotic risk and clinical evidence of bleeding. UFH was not used in patients with severe thrombocytopenia or suspected or confirmed heparin-induced thrombocytopenia.

2. Regional citrate anticoagulation

Regional citrate anticoagulation (RCA) was performed using 4% trisodium citrate (136 mmol/L), infused pre-filter at a rate proportional to blood flow. For CVVHD, the initial citrate dose was 3.0 mmol per litre of blood. Calcium was replaced separately through the venous return line using a calcium chloride solution containing 97 mmol/L calcium, with an initial calcium dose of 2.0 mmol per litre of effluent. Calcium-free Ci-Ca® Dialysate K2/K4 PLUS was used as dialysate.

Post-filter ionized calcium was used to titrate citrate delivery, whereas systemic ionized calcium was used to titrate calcium replacement. The first post-filter ionized calcium measurement was obtained approximately 5-10 min after treatment initiation to verify correct circuit and solution setup. Thereafter, post-filter and systemic ionized calcium were monitored every 6 h.

For CVVHD, citrate delivery was adjusted according to post-filter ionized calcium as follows: >0.45 mmol/L, +0.3 mmol/L; 0.41-0.45 mmol/L, +0.2 mmol/L; 0.35-0.40 mmol/L, +0.1 mmol/L; 0.25-0.34 mmol/L, no change; 0.20-0.24 mmol/L, -0.1 mmol/L; 0.15-0.19 mmol/L, -0.2 mmol/L; and <0.15 mmol/L, -0.3 mmol/L.

Calcium replacement was adjusted according to systemic ionized calcium as follows: >1.45 mmol/L, -0.4 mmol/L; 1.31-1.45 mmol/L, -0.3 mmol/L; 1.21-1.30 mmol/L, -0.2 mmol/L; 1.12-1.20 mmol/L, no change; 1.05-1.11 mmol/L, +0.2 mmol/L; 0.95-1.04 mmol/L, +0.4 mmol/L; and <0.95 mmol/L, +0.6 mmol/L.

Routine monitoring during RCA additionally included arterial blood gas analysis before CRRT initiation and every 6 h, general biochemistry including total calcium, magnesium, phosphorus and liver parameters at baseline and at least every 24 h, and daily hematological and coagulation assessment.
